# Leach’s storm-petrel (*Hydrobates leucorhous*), a long-lived seabird shows flexible, condition-dependent, feeding strategies in response to poor chick condition

**DOI:** 10.1186/s12862-024-02273-8

**Published:** 2024-07-01

**Authors:** Benjamin D. Haussmann, Kayla E. Lichtner, Robert A. Mauck, Mark F. Haussmann

**Affiliations:** 1 Department of Biology, University of Nebraska at Omaha, Omaha, NE 68182 USA; 2 Department of Biology, Bucknell University, Lewisburg, PA 17837 USA; 3 Department of Biology, Kenyon College, Gambier, OH 43022 USA

**Keywords:** Feeding behavior, Foraging behavior, Foraging strategy, Life history evolution, Seabird

## Abstract

**Background:**

Parent-offspring conflict represents the sensitive balance of resource allocation between self-maintenance and reproduction. Two strategies have been proposed to better understand how species manage this conflict. In fixed-level feeding behavior, parents feed offspring consistent quantities of food; while flexible feeding shows plasticity in parental allocation based on offspring need. Life-history theory predicts that parents of long-lived species prioritize their survival and may favor the fixed-level hypothesis to maximize lifetime reproductive success. In this study, we highlight the natural variation of parent-offspring allocation strategies within a unique population of Leach’s storm-petrels (*Hydrobates leucorhous*), and through month-long food supplementation and restriction manipulations, we investigate how chick condition affects parental provisioning during the chick-rearing period of reproduction.

**Results:**

We show that the parents upregulated chick feeding frequency of nutritionally deprived chicks, resulting in a larger total amount of food delivered during the study period. Additionally, the proportion of nights when both parents fed was highest in restricted chicks, and the proportion of nights when neither parents fed was lowest in restricted chicks, suggesting that storm-petrel parents shorten their foraging bouts to deliver food more often when their chicks are in relatively poor condition.

**Conclusions:**

Our results support that Leach’s storm-petrels use a flexible-level feeding strategy, suggesting that parents can assess offspring condition, and respond by feeding chicks at higher frequencies. These data provide insight on how a long-lived seabird balances its own energetic demands with that of their offspring during the reproductive period.

**Supplementary Information:**

The online version contains supplementary material available at 10.1186/s12862-024-02273-8.

## Background

During reproduction, parents must strike a balance between energy investment to self maintenance and reproductive success [1]. A stochastic environment may further complicate this parent offspring conflict as foraging conditions can vary widely across the season and among years [2]. Two theoretical frameworks of food allocation to offspring, which consider the condition of parents and offspring as well as the environment are broadly defined as the fixed-level and flexible-level allocation strategies. The fixed-level strategy suggests that evolutionary processes, such as prioritizing self maintenance or maximizing lifetime reproduction, shape parental food investment of a fixed quantity, and this strategy would be useful in areas with a consistent food source [3]. Conversely, the flexible-level strategy suggests that parents can assess offspring nutritional conditions, and adjust parental investment to maximize current reproductive success [4]. This strategy would be advantageous in areas where food quantity or quality fluctuates [5].

In long lived species, life-history theory predicts that parents prioritize allocation of food resources to themselves, while parents of short lived species prioritize their offspring [6, 7]. One would expect that long-lived species may favor the fixed-level hypothesis as individuals must regulate current offspring investment in the context of maximizing lifetime reproductive success [8–10]. However, there is conflicting literature regarding this assertion as different studies suggest that long lived species may use either allocation strategy. An illustrative example of this can be found with Cory’s Shearwater *(Calonectris borealis)* in which two different studies performed similar short-term food restriction manipulation experiments. While one study reported no compensatory parental response to poor chick condition [11], the other study reported that parents provided larger meals and fed at higher frequencies in response to poor chick condition [12]. This lack of agreement calls for further investigation to better understand parental allocation patterns in long-lived species.

Procellariiformes are long lived pelagic seabirds that can serve as an excellent model to better understand this parent-offspring conflict in resource allocation [13]. Procellariiformes exhibit biparental care during reproduction [14], and typically invest in a single offspring each year [15]. Once offspring hatch, parents spend most of their time foraging, and only spend brief periods in the burrows to feed chicks. These foraging trips can be energetically expensive as parents must often travel long distances to acquire high quality food which they share with their offspring [16]. However, food sources can vary in both quantity and quality within seasons and across years, presenting a challenge for parents [17]. Taken together, this provides an ideal system to explore the fixed and flexible-level allocation strategies.

The aims of this study were to (1) record the natural variation in feeding behavior in Leach’s storm petrels (*Hydrobates leucorhous*) over the course of their breeding season and (2) in the following year, use an experimental manipulation to shed light on the allocation strategy of long-lived species. Previous studies focus on short-term food restriction, but these may fail to capture whether or not parents shift between strategies based on environmental context. Thus, we hypothesize that long-term monitoring and manipulations may better capture if parents shift to a flexible-level allocation strategy when periods of food deprivation persist.

## Methods

### Study site

Birds were sampled from a breeding colony of approximately 20,000 pairs of Leach’s storm-petrels at the Bowdoin College Scientific Station on Kent Island, New Brunswick, Canada (66°45’ W, 44°35’ N) which has been monitored annually since 1953 [18]. We studied the breeding behavior of Leach’s storm-petrels from June through October in 2006 and 2007. Our study population consisted of 311 burrows, with approximately 170 burrows occupied by nesting pairs each year. Beginning on 30 May in both years, we monitored all burrows daily to determine burrow occupancies and lay dates by briefly reaching into burrows to determine the presence of an egg. Once an egg was detected, we discontinued daily monitoring for 38 days to minimize disturbance before hatching. Each year from the subset of 170 occupied burrows, we arbitrarily chose 60 burrows to monitor which were dispersed across the entire study site. After eggs were in study burrows for 38 days, burrows were checked daily for chicks. Hatch date was designated as day 0 of age, and we did not disturb chicks again until day 4 of age, when parents generally stop brooding the newly hatched chick. In Leach’s storm-petrels, both sexes share the work of the 43-day incubation period and the 66-day chick-rearing period [18] and this study focused on the latter period. During the chick-rearing period, each nestling is fed during brief nocturnal visits by its parents [19], and each adult petrel typically returns to the nest every second or third night until the chick fledges [20]. Thus, on any given night a chick may be fed by both parents, one parent, or no parent. Chick mass is therefore highly variable day to day [15, 20, 21] and chick mass closely approximates food provisioning by adults [18].

#### Year one (2006): natural variation in Leach’s storm-petrel parental feeding behavior

During the 2006 breeding season, we measured chick mass daily from 4 to 42 days of age. These masses were used to define parental feeding behavior as described below.

Starting at 4 days of age and until 42 days of age, the mass of all study chicks was taken once every 24 h between 1000 and 1100 h. We used this daily mass measure to calculate 24 h chick mass changes which serves as a proxy for total meal size delivered on the preceding night. For simplicity, we define 24 h chick mass change as “meal size”.

We assessed food delivery in two ways: feeding frequency (proportion of chicks receiving or not receiving a feeding visit each night) and meal size (total amount of food received overnight by the chick on nights when food was delivered). In this study, adults were not able to be fitted with PIT tags to determine identity and whether one or both parents visited the burrow on a night when chicks were fed. Therefore, we estimated meal size based on previous studies done in this colony [10, 22]. If parents did not feed the chicks on a night, we referred to this as “no feed”, and this resulted in mass losses. We used the terms ‘‘single feed’’ and ‘‘double feed’’ to refer to the estimated amount of food delivered by either a single parent on a night, or by both parents on a night, respectively.

##### No feed nights

Leach’s storm-petrel chicks between 10 and 50 days of age metabolize an average of 2 g of body mass every 24 h [10]. Given this, if a chick lost ≥ 2 g of mass over a 24 h period it was assumed they were not fed by parents during the previous night.

##### Single feed night

Previous work done in the colony reported that meal size during a visit from one parent (single feed) averaged 8.7 g [19] or 8 g [22]. Based on the average loss of 2 g due to basal metabolism, any chick’s mass which was within the range of -2 g to 6 g compared to the previous day’s mass was assumed to be fed by a single parent [19].

##### Double feed night

Any chick that gained more than 6 g from the previous day’s mass was assumed to be visited and fed by both parents [19].

We acknowledge that while there will be some error using these guidelines, they closely agreed with previous work done by us in this colony that used PIT tag readers to confirm single feed or double feed nights ( [23]; Supplemental Material).

#### Year two (2007): manipulating food provisioning of Leach’s storm-petrel parents

In 2007, we measured chick masses daily as described above which were used to define parental feeding behavior. In addition, we performed feeding manipulations on chicks to better assess parental feeding behavior strategies. Active burrows were identified in the same way as 2006, however, once eggs were detected, burrows were placed into one of the three groups using a stratified random design which accounted for lay date (*n* = 20 each): control, supplemented, or restricted.

##### Control chicks

In 2007, the control chicks were treated identically to chicks in 2006.

##### Supplemented chicks

Because storm-petrel chicks gain mass irregularly over the nestling period [21], the supplemented group was included to determine the effects of reducing food irregularity. We manipulated this group to experimentally recreate chicks that had more regular feedings (the top 10% of chicks in 2006). Supplemented chicks were treated identically to control chicks, except that if the chicks gained less than 1 g in mass from their previous day’s body mass (indicative of minimal or no parental food delivery during the night), they were supplemented with 2 g of food. The supplementary food consisted of a mixture of 1:3 olive oil to krill mixture (krill mixture is 1:5 krill to water), homogenized to a smooth, fluid paste in a food blender [10, 24]. This 2 g of supplemented food provides energy that is approximately equivalent to the total daily metabolized energy of a large chick [10]. The mixture was administered by intubation from a small piece of tygon tubing connected to a disposable plastic syringe into the esophagus. Control and restricted chicks were given a sham feeding every other day in which the feeding apparatus was placed into their esophagus but no food was delivered to control for any potential stress of the supplemental feeding. Once chicks reached 42 days of age, they no longer received any supplemental or sham feedings.

##### Restricted chicks

We manipulated chicks in this group to experimentally recreate chicks that had the least access to food in 2006 (the bottom 10% of chicks). Restricted chicks were treated the same as control chicks however, these chicks only received half of the feedings from their parents. We accomplished this by splitting the twenty chicks in this group into matched pairs of restricted-treatment chicks, with each member of the pair being a similar age (mean difference in age = 1.4 ± 0.4 days). In each matched pair, one of the chick’s burrows was randomly selected as the active burrow, which was the burrow that always contained one of the chicks for the rest of the nestling period. The chick in the pair that was originally from that active burrow remained there, while its matched restricted chick was placed in an artificial burrow. We had ten artificial burrows within the study site. Artificial burrows contained normal nest material and were within the range of temperature and humidity as natural burrows, but were blocked from visits by adult storm-petrels. Chicks within a matched pair were alternated between the active and artificial burrows whenever the chick in the active burrow was fed by an adult (this could be determined by the change in mass from the chick’s previous day’s body mass). Previous work in this colony reported that parents do not appear to distinguish between their own chick and the exchanged chick as food delivery is similar between the two [22]. By switching the chicks within a matched pair in this way, we ensured that the restricted chicks received half of the normal amount of parental feeding bouts, with the result that parents returning to the nest are always confronted with a hungry chick.

We monitored restricted chicks for altered behavior, and if the chick appeared cold and torpid we supplemented them with 5 g of food (in the way supplemented chicks were fed as described above). This was not a common occurrence and on average each restricted chick was supplemented 0.6 ± 0.8 (mean ± 95% CI) times over the nestling period. Once restricted chicks reached 42 days of age we no longer collected data for this study, but we continued to switch the members of a pair between the active and artificial burrow. However, we supplemented the chick in the artificial burrow with the same amount of food as was gained by the chick in the active burrow from the previous night so now both chicks were receiving a normal amount of food. All restricted chicks fledged, suggesting that this manipulation did not affect nestling mortality.

### Statistics

We examined parental feeding behavior with two measures: feeding frequency and meal size. For feeding frequencies, we focused on the proportion of nights that chicks were visited by one parent, two parents, or no parent for each burrow. To do this, we ran generalized linear mixed models (GLMMs) using JMP Pro software (v.17.1.0, SAS Institute Inc. 2023, Cary, NC, USA). If data were normally distributed we fitted the models with normal distributions and identity functions, and if they were not, we used binomial distributions and a logit link function. For 2006, feeding frequency was included as the dependent variable, and number of parents (zero, one, or two) as a fixed effect. For 2007, we also included treatment (control, restricted, supplemented) and the interaction of the number of parents and treatment as fixed effects. To compare years, all chicks from 2006 were compared to the control chicks in 2007, and year, number of parents, as well as their interaction were included as fixed effects. We included ‘individual’ as a random factor to control for the non-independence of data due to repeated measurements on the same individuals. We carried out post hoc comparisons using Tukey HSD tests.

For meal size, we included all meals delivered to chicks from 4 to 42 days of age for each burrow. We ran linear mixed effects models (LMM) using JMP Pro software (v.17.1.0, SAS Institute Inc. 2023, Cary, NC, USA). For every model, we checked for homogeneity of variances (Levene’s test), and for normality of residuals (Kolmogorov–Smirnov test). We fitted the models with a Gaussian error distribution and an identity link function. For 2006, meal size was included as the dependent variable, and number of parents (zero, one, or two) as a fixed effect. For 2007, we also included treatment (control, restricted, supplemented) and the interaction of the number of parents and treatment as fixed effects. To compare years, all chicks from 2006 were compared to the control chicks in 2007, and year, number of parents, as well as their interaction were included as fixed effects. We included ‘individual’ as a random factor to control for the non-independence of data due to repeated measurements on the same individuals. We carried out post hoc comparisons using Tukey HSD tests.

## Results

### Natural variation in storm-petrel feeding behavior

**Fig. 1:**
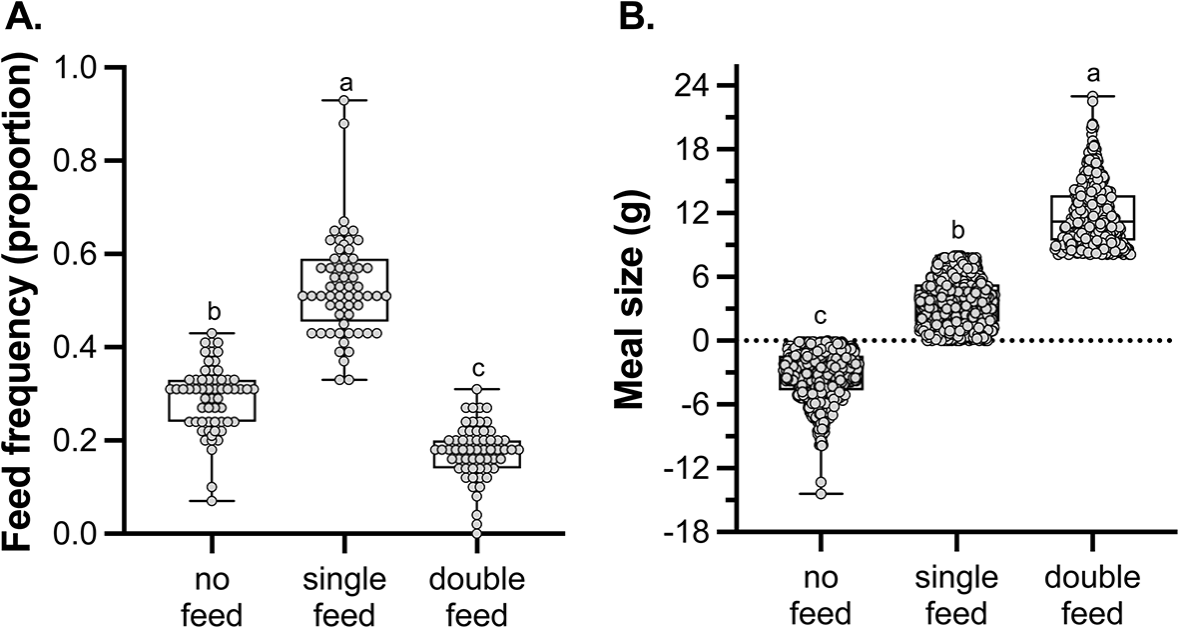
The proportion (**A**) and meal size (**B**) of no feed, single feed, and double feed nights in Leach’s storm-petrels in 2006. Scatter plots are shown, and for feeding frequencies (**A**) each of 60 chicks are represented by a point for each parental feed category (no, single, double), while for meal size (**B**) every night of the study period for each chick is represented by a point for each parental feed category (no, single, double). Bar whisker plots show median and variation. Different letters denote differences among feed groups calculated by post hoc comparisons using Tukey HSD tests

In 2006 between day 4 and day 42, the average proportion of chicks receiving at least one feeding visit per night was 0.68 ± 0.06 (mean ± 95% CI), and the average amount of food delivered per night was 5.7 ± 0. 06 g (mean ± 95% CI). On any given night in a burrow, single feeds occurred most often, followed by nights where no parents fed, and double feeds occurred the least frequently (𝛘^2^_1_=17.6; *p* < 0.0001; Fig. 1A). Meal size was predictably larger in double feeds compared to single feeds, and when not fed, chicks lost an average of 3.2 ± 2.2 g (mean ± 95% CI) of mass a night (F_2,2752.5_ = 6750.1; *p* < 0.0001; Fig. 1B).

### Food manipulation effects on storm-petrel feeding behavior

**Fig. 2:**
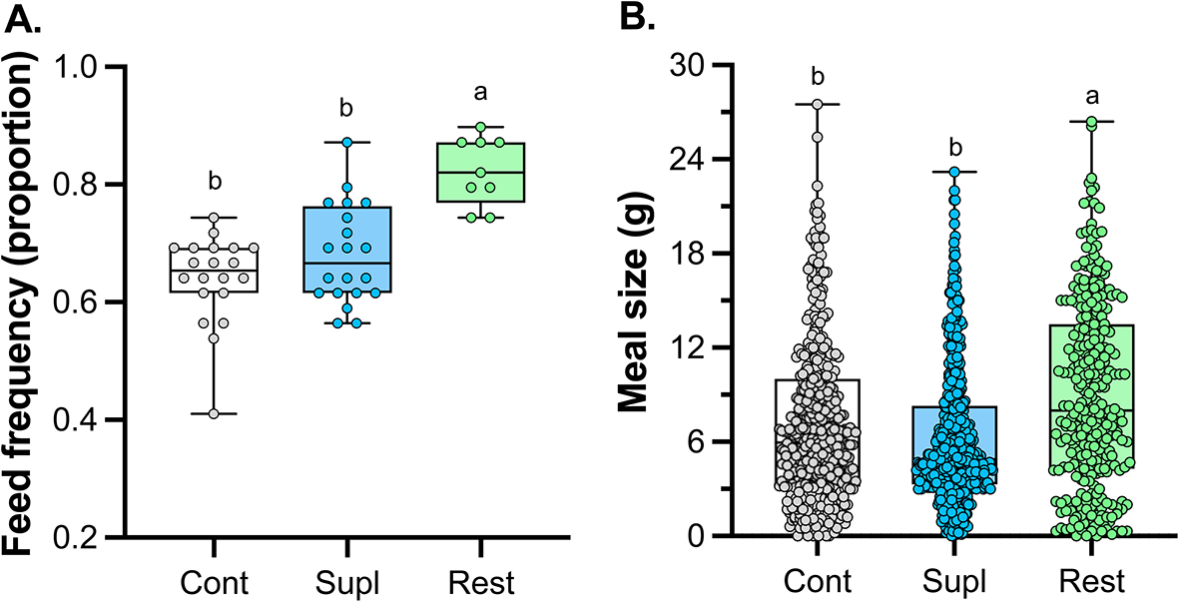
The proportion (**A**) and meal size (**B**) for control (cont; gray symbol, white box), supplemented (supl; blue symbol, blue box) and restricted (rest; green symbol, green box) treatment groups in Leach’s storm-petrels in 2007. Scatter plots are shown, and for feeding frequencies (**A**) the number of burrows is represented by a point for each treatment, while for meal size (**B**) every night of the study period for each chicks is represented by a point for each treatment. Bar whisker plots show median and variation. Different letters denote differences among treatment groups calculated by post hoc comparisons using Tukey HSD tests

In 2007 between day 4 and day 42, the average proportion of chicks receiving at least one feeding visit per night varied among the three treatments (𝛘^2^_1_ = 28.3; *p* < 0.0001; Fig. 2A). Specifically, restricted chicks were fed at a higher frequency than either control or supplemented chicks (Tukey HSD, *p* < 0.0001), which had similar feeding frequencies (Tukey HSD, *p* = 0.2). The average amount of food delivered per night also varied among the three treatments (F_2,41_=17.2; *p* < 0.0001; Fig. 2B). Specifically, restricted chicks were fed larger amounts of food on average when parents visited compared to control or supplemented chicks (Tukey HSD, *p* < 0.0009), which had similar meal sizes (Tukey HSD, *p* = 0.08). Importantly these analyses do not account for the number of parents feeding on any given night.

**Fig. 3:**
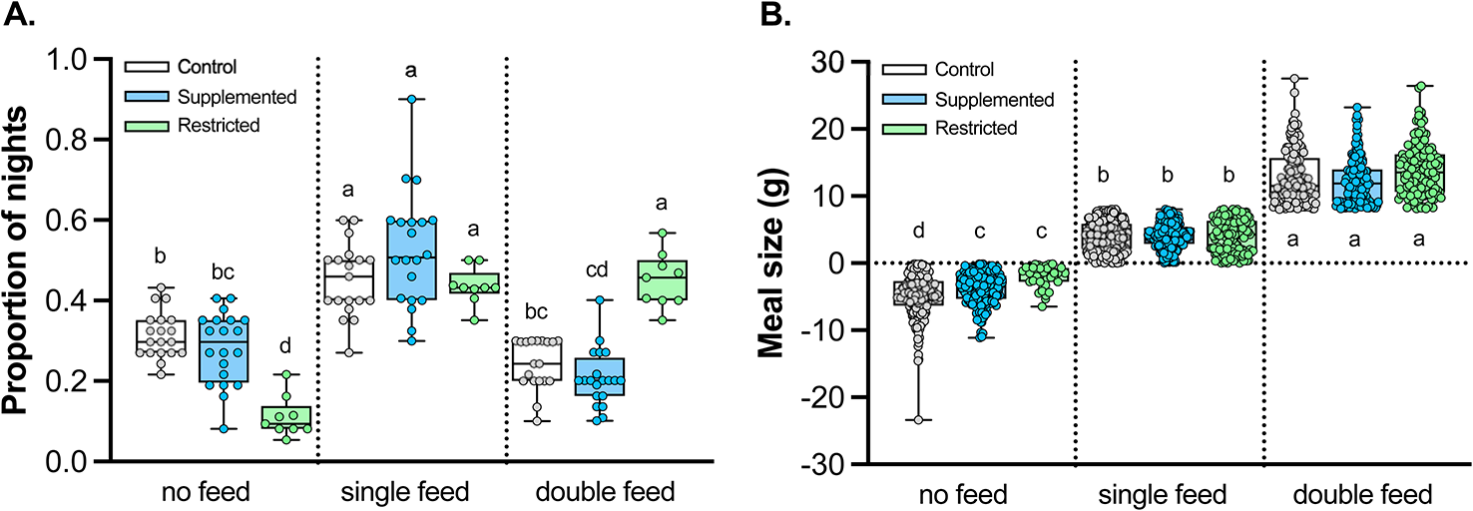
The proportion (**A**) and meal size (**B**) for control (cont; gray symbol, white box), supplemented (supl; blue symbol, blue box) and restricted (rest; green symbol, green box) treatment groups in no feed, single feed, and double feed nights in Leach’s storm-petrels in 2007. Scatter plots are shown, and for feeding frequencies (**A**) the number of burrows is represented by a point for each treatment, while for meal size (**B**) every night of the study period for each chicks is represented by a point for each treatment. Bar whisker plots show median and variation. Different letters denote differences among feed groups and treatments calculated by post hoc comparisons using Tukey HSD tests. If a group has two letters, for example ‘bc’ it designates that group is similar to any other group that has either a ‘b’ or a ‘c’ letter

When the number of parents were included in the analyses, there was a significant treatment by number of parents interaction (𝛘^2^_2_ = 79.7; *p* < 0.0001; Fig. 3A). Specifically, while there was a similar proportion of single feed nights among the three groups (0.48 ± 0.07 of nights, mean ± 95% CI; Tukey HSD, *p* > 0.2), the proportion of double feed nights was higher and the proportion of no feed nights was lower by parents of restricted chicks, compared to the parents of the control and supplemented chicks (all Tukey HSD, *p* < 0.0001; Fig. 3A). The size of meals on single feed and double feed nights did not differ among the three treatments (all Tukey HSD, *p* > 0.4, Fig. 3B), though control chicks lost slightly more mass on no feed nights than the supplemented or restricted chicks (F_4,1711.2_ = 9.7; *p* < 0.0001; all Tukey HSD, *p* < 0.03).

### Comparison of 2006 and unmanipulated 2007 feeding behavior

There was a significant year by number of feeding parents interaction revealing that the average proportion of chicks being fed by neither, one, or both parents each night differed between years (𝛘^2^_2_ = 24.7; *p* < 0.0001). Specifically, while there was a similar proportion of no feed nights between years (Tukey HSD, *p* = 0.8), in 2006 there was a higher proportion of single feed nights (Tukey HSD, *p* < 0.0001), and in 2007 there was a higher proportion of double feed nights by the control chicks (Tukey HSD, *p* = 0.009). In addition, there was also a significant year by number of feeding parents interaction showing that meal size also differed depending on the number of parents visiting on a night between the two years (F_2,3521.8_ = 48.1; *p* < 0.0001). Specifically, while meal size did not differ for single feed or double feed nights between years (All Tukey HSD, *p* > 0.07), control chicks in 2007 lost more mass following nights that parents did not feed compared to 2006 chicks (Tukey HSD, *p* < 0.0001).

## Discussion

Our study explored parent-offspring allocation strategies in Leach’s storm-petrels, both by describing natural variation in parental feeding behavior and by investigating how manipulating chick access to food affected parental feeding behavior. In the unmanipulated year, we found that chicks were fed by a single parent about half of the nights, by both of the parents a quarter of the nights, and not fed by either parent on the remaining quarter of nights. In the manipulated year, supplemented chicks that were experimentally given additional food did not affect parental feeding behavior. However, when the chicks had their access to food limited by half, the proportion of single parent feed nights remained similar to the unmanipulated year, but the proportion of double parent feed nights increased substantially and the proportion of nights that no parents fed fell close to zero. These data provide strong support for a flexible-level allocation strategy in this long-lived seabird.

While parents of restricted chicks altered their feeding behavior by increasing the frequency of their feeding visits, there was no change to the size of the meal delivered. Specifically, the size of meals delivered to chicks did not differ between the unmanipulated year and the manipulated year among the three treatment groups. Taken together, while the parents of restricted chicks did not provide larger individual meals, by altering their feeding frequency, they provided a larger total amount of food to the chicks over the course of the manipulation. Other studies in pelagic seabirds agree with our finding of a flexible-level feeding strategy [5, 8, 25–27]. For example, a study exploring natural variation in Dovekie (*Alle alle*) feeding behavior found that parents changed the number of feeding visits they made depending on food quality and abundance [17]. Another study which used a manipulation to restrict the amount of food Scopoli’s shearwater (*Calonectris diomedea*) chicks received over a six day period reported that parents began delivering larger quantities of food to these restricted chicks [12].

While our results agree with other papers which found support for a flexible-level parental allocation strategy, other studies find support for the fixed-level parental allocation strategy [11, 15, 22, 28], illustrating a lack of consensus on parental allocation strategies. Interestingly, some of these other studies were done on the same population of Leach’s storm-petrels nesting on Kent Island. For example, Ricklefs [22], who performed a similar restriction manipulation to the current study, found that storm-petrel parents did not respond to chick undernourishment by increasing feeding rate. However, this study was only performed for 6 days. And, in another study on Scopoli’s Shearwater, Hammer and Hill [28] reported that neither meal size nor feeding frequency were related to chick body condition.

One possible way to reconcile these two seemingly conflicting bodies of literature is that both fixed and flexible-level allocation strategies exist, but within populations these strategies may shift back-and-forth in a context-dependent fashion [8]. Seabirds live in an unpredictable environment which can vary widely both within [29–31] and among years [2, 17]. This uncertain environment may favor breeding strategies where parental allocation can change and adjust according to the prevailing environmental conditions and food availability [9]. Interestingly, studies that focused on shorter periods of parental feeding behavior, or for only one season, were more likely to find support for a fixed-level allocation strategy. While studies that were across seasons were more likely to find support for a flexible-level allocation strategy. Given the stochastic nature of seabird environments, studies focusing on a longer period may be more likely to capture that variability, and thereby a shift in parental feeding strategies. Unlike previous studies that followed parental feeding behavior for relatively short periods of one to four days [12, 26, 27, 32], or for up to a week [5, 17, 22, 28, 33], our study period lasted for 38 days in each of the two seasons, which is a large proportion (∼58%) of the chick rearing period. Interestingly, 2006 had a higher proportion of single feed nights, while 2007 had a higher proportion of double feed nights. This suggests a potential difference in food quality or abundance between the two years which resulted in parents altering their feeding behavior to feed more often in 2007. This is not surprising given the broad interannual variation in food quality in marine systems in general, and in our Leach’s storm-petrel study system in particular [34]. The increase in nights where both parents visited the burrow suggests that 2007 may have been a more challenging year compared to 2006. In agreement with this, chicks had larger 24-hour mass losses on days after a night where no parent visited the burrow to feed chicks, suggesting those chicks may have had to use a larger proportion of their food reserves to fuel their metabolism between feedings. It is important to note, that while there were differences in 24-hour mass loss between 2006 and 2007, when comparing data to 2008, a year when PIT tags were utilized to that allowed us to measure how many parents entered a burrow each night, the number of parents visiting each night was similar across all three years (Supplemental figure).

Our finding that parents modified feeding frequency, but not meal size is reported in other studies as well [17, 35]. This suggests that parents may be feeding until their food payload reaches a physical limit [11]. Alternatively, parents may only be willing to feed chicks so much of their food store to protect their own nutritional demands. Leach’s storm-petrels, like other Procellariiforms, have high adult survival, and the single chick reared each year represents a small portion of their lifetime reproductive success, so parents prioritize investment to safeguard themselves over the needs of their chicks [15]. For example, a study on Antarctic Petrels *(Thalassoica antarctica)* showed parents who had a poor body condition fed less food to offspring [5]. In another example, a recent study on Dovekies by Kidwa et al. [32]) found that parents dosed with corticosterone, a hormone that is reliable indicator of poor condition [36], fed their chicks less food compared to parents with unmanipulated corticosterone levels [32]. Taken together, this suggests that Procellariiform parents base decisions on allocations to their offspring by assessing their needs. In support of this, studies that supplemented chicks daily with large amounts of food found that parents delivered less food to their offspring and, in turn, kept more for themselves [24, 33]. While we did not find this in our study, the food supplemented to chicks, was only 2 g, and more so, chicks were only supplemented following nights that parents did not feed. This was intended to ensure no mass loss occurred, but this limited amount does not appear to have been enough to alter parental feeding behavior.

The amount of food an adult can carry is limited. In addition, over the course of a foraging bout, parents must use some of the food they gather to fuel the demanding cost of flight [37]. Thus, the food hauled back to their offspring does not represent the total sum of the food gathered during the foraging trip, and during longer foraging trips a larger total amount of food is gathered and used by the parent [38]. Therefore, if storm-petrel parents shift to feeding their offspring similarly sized meals more frequently, they may have less food for themselves. In agreement with this, other studies in Procellariformes have observed a dual foraging strategy, where adults will travel short distances to forage food for their young, and longer distances to forage for themselves [16, 38]. These extended foraging trips enable parents to acquire, process, and excrete food needed for self-maintenance completely before obtaining more food to deliver to their offspring [39, 40]. While this dual feeding strategy has not been directly observed in Leach’s storm-petrels, parents traveling shorter distances would allow for more frequent visits to burrows. However, this also suggests that the parents of restricted chicks may have been relatively resource limited themselves, which could result in some physiological costs. Future work should attempt to quantify these costs and determine if they affect lifetime reproductive success.

## Conclusions

In conclusion, our results support the flexible effort hypothesis, and suggest that Leach’s storm-petrels are able to adjust their reproductive effort by feeding chicks in poor body condition more frequently. The degree to which parents will increase their investment into their offspring before abandoning the breeding attempt is not known and presumably depends on the quantity and quality of food available as well as the parent’s prospects of survival. In addition, this is likely to differ among individuals within the population, and is deserving of further long-term manipulative studies to better understand how parents of long-lived species optimize current and future reproductive success in unpredictable environments.

### Electronic supplementary material

Below is the link to the electronic supplementary material.

Supplementary Material 1

## Data Availability

The datasets used and/or analyzed during the current study are available from the corresponding author on reasonable request.
